# 

**Published:** 1853-05

**Authors:** 


No. XII. MAY. Vol. VIII.

### BUFFALO

Philadelphia

### MEDICAL JOURNAL

AND

### MONTHLY REVIEW

OF

### MEDICAL AND SURGICAL SCIENCE.

EDITED BY AUSTIN FLINT, M. D.

BUFFALO:
PUBLISHED BY JEWETT, THOMAS & CO.
Commercial Advertiser Buildings.

1853

*Price* $2.50 *per annum . . . . in Advance.*

